# Supplementary material for: Assessing women’s preferences towards tests that may reveal uncertain results from prenatal genomic testing: Development of attributes for a discrete choice experiment, using a mixed-methods design
Source: PLoS One. 2022 Jan 28;17(1):e0261898. doi: 10.1371/journal.pone.0261898 (PMC8797177; doi:10.1371/journal.pone.0261898)
Supplement: S1 Fig — (DOCX) [file pone.0261898.s001.docx]

**Search terms used for systematic review**

[pregnant women OR women OR prenatal OR parent* OR fetal OR foetal]

AND

[chromosom* microarray OR CMA OR arraycgh OR array* OR genome sequenc* OR exome sequenc* OR genome-wide OR microdeletion* OR microduplication* OR submicroscopic OR subchromosom*] AND

[experience* OR view* OR attitude* OR preference* OR perception* OR choice* OR choos]

**Search terms used for review of other published DCEs**

[discrete choice experiment OR conjoint analysis]

AND

[exome sequencing OR genome sequencing OR microarray OR CMA OR incidental finding OR secondary finding]
