# Supplementary material for: Assessing women’s preferences towards tests that may reveal uncertain results from prenatal genomic testing: Development of attributes for a discrete choice experiment, using a mixed-methods design
Source: PLoS One. 2022 Jan 28;17(1):e0261898. doi: 10.1371/journal.pone.0261898 (PMC8797177; doi:10.1371/journal.pone.0261898)
Supplement: S2 Fig — (DOCX) [file pone.0261898.s002.docx]

# **Dealing with uncertainty in prenatal genomics**

##### **Questions for patients**

## Introduction

- Thank you for agreeing to participate in this interview
- Uncertain results may arise for all sorts of reasons in prenatal testing. It may be a result for which the effect on the baby is uncertain because the features of the condition vary a lot. Or a result may be uncertain because there is a chance, rather than a certainty, that the baby will be affected by a condition, for example a 1 in 5 chance or 20% chance. Or it may be uncertain that a gene change that has been found is the cause of symptoms seen in the baby – especially if it is a gene change that has not been seen before.
- We are interested in understanding your views and experience of receiving uncertain results in pregnancy, both before and after you received these results.

1. Can you start by telling me about what happened during your pregnancy, in particular the tests you had and when you received results that had some element of uncertainty.

“Thank you for sharing that with me. I’m now going to ask you some questions to understand more about the different stages you went through..”

###### [Questions about ultrasound screening]

1. What led to your decision to have ultrasound screening?
   1. What were you hoping to get out of the screening test?
   2. Who discussed the test with you?
   3. What were you told about the screening test? Were some of the limitations or potential uncertainties of the test you remember being discussed? [probe: about the possibility to find something uncertain?]
   4. Did you feel you had adequate information about the test to make an informed choice about having the screening test?
2. What were the findings from the screening test?
   1. Who explained the results to you?
   2. How long did that explanation take?

[If uncertain results were found at this stage]

1. How would you describe the way the ‘uncertain’ results were discussed with you? [probe: Were they easy to understand? Complex? Did you feel you had a clear understanding of why the result was difficult to interpret?]
2. What was it like to receive an uncertain result?
3. Can you describe what it was like emotionally (prompt: frustrating, worrying, loss of control)
4. What were the practical implications of the uncertain result?
   1. Did you have any additional appointments or further discussions with any healthcare providers (doctor, midwife, genetic counsellor)?
5. Were you given different opinions about the result from different health professionals?
6. Did you have any emotional support during this time? If so, from whom? (Other health professionals, Friends/family, Support groups)
7. What happened next? Were you offered invasive testing?
8. Did you decide to have invasive testing?
   1. If no, can you describe your reasons for declining?

## [Women that went on to have invasive testing]

1. What led to your decision to have invasive testing?
2. How long did you wait for the test results?
3. What were the results from the invasive test?
   - 1. Did you consider the result uncertain?
     2. Was the uncertainty resolved or did the uncertainty still remain?
4. What did you decide to do? (continue or terminate the pregnancy?)
5. Was the uncertainty resolved later on? (In the pregnancy? Following the birth?)
6. [If continued pregnancy] Did the uncertainty have any impact on the rest of the pregnancy (prompt: emotional, practical e.g. more tests, where baby was born, what happened when baby was born)

[All participants]

1. Has this experience impacted your thoughts about having another pregnancy?
   1. If so, how?
   2. Would you have prenatal screening/testing?
   3. Is there anything you would do differently?
   4. Would you want health professionals to tell you about a result if there was some uncertainty associated with it?
2. [For those who have since gone on to have another pregnancy] Did your experience impact your next pregnancy in any way? If yes, how?

[General thoughts about providing uncertain results during pregnancy]

1. What do you think are the main factors that made the uncertainty **difficult to deal with**? [prompt: diagnosis, prognosis, been unprepared for uncertain results, poor communication, conflicting information from different sources]
2. In hindsight, what do you think might have made the experience of receiving uncertain **results ‘easier’ to deal with**? [prompt: more time with health professional, more emotional support, better communication]
   1. What kind of support is needed during pregnancy following an uncertain result?
   2. Are there any other things you found important when dealing with the result?

21. Recently, new tests have been developed in pregnancy that can look much more closely at the DNA of a fetus. Doctors and midwives are now beginning to offer these kinds of tests in pregnancies when something unusual about the fetus is seen on the ultrasound. However, one of the concerns with these new tests is whether we will more frequently come across results that have some element of uncertainty and if we do, which of those results we should and should not feed back to patients. We would like to ask you what you think.

1. [VUS] Sometimes we find a change in a gene but we cannot be 100% certain that the change we find is the cause the health problem in the fetus. In those circumstances, do you think we should tell the patient what was found? What level of certainty should the doctor have before they tell the patient?
2. [Reduced penetrance] Sometimes we find a change in a gene but not everyone with that change will go on to get the condition. Do you think we should report these to parents? In what circumstances do you think we shouldn’t report them?
3. [Variable expression] Sometimes we find a change in a gene that causes a health condition, but the symptoms of that condition vary considerably i.e. some people may have very mild symptoms while others are more severely affected. Do you think we should report these to parents? In what circumstances do you think we shouldn’t report them?
4. [Diagnostic yield] Even though these new tests look very closely at the DNA of the baby, we are not always able to find an answer to the problems that have been seen on the scan. If the test was invasive, what do you consider to be a reasonable ‘pick-up’ rate in term of finding an answer, before you agreed to take the test? Is there any ‘pick-up’ rate when it would not be appropriate to offer invasive testing?
5. [Secondary findings] Sometimes we find changes in a gene that cause a health condition which are unrelated to the original reason for testing. Do you think these should be reported to parents? Are there certain findings that we shouldn’t report?
