# Supplementary material for: Assessing women’s preferences towards tests that may reveal uncertain results from prenatal genomic testing: Development of attributes for a discrete choice experiment, using a mixed-methods design
Source: PLoS One. 2022 Jan 28;17(1):e0261898. doi: 10.1371/journal.pone.0261898 (PMC8797177; doi:10.1371/journal.pone.0261898)
Supplement: S3 Fig — (DOCX) [file pone.0261898.s003.docx]

# **Dealing with uncertainty in prenatal genomics**

##### **Questions for clinical scientist/ laboratory geneticist**

## General

1. In what circumstances would pregnant women be offered CMA in your hospital?
2. Are women being offered WES prenatally in your hospital and if so, in what circumstances would it be offered? Prompt: is it only in research setting or is it being offered clinically?
3. Do women pay of these tests themselves or are they covered e.g by national health service or insurance system? If they pay themselves what are the costs for CMA and WES?

Sources of uncertainty

1. What are the main uncertainties that *you* come across with CMA/WES?
2. In your experience have you come across any of the following *types* of uncertainty and if so can you give any examples?
3. uncertainty related to gene-disease correlations;
4. uncertainty about how a genetic anomaly with a well-known postnatal phenotype presents prenatally,
5. variants of uncertain clinical significance,
6. penetrance and expression variations,
7. making an unexpected diagnosis not related to clinical phenotype
8. secondary/incidental findings,
9. technical validity of a call (for which further validation may be required e.g. false positives, false negatives, depth of the read not sufficient to be certain of results)
10. possible incomplete results e.g. 1 autosomal recessive variant compatible with the fetal phenotype, but no second mutation identified)
11. Have you experienced any other type of uncertainty in your day-to-day work?
12. Thinking about the types of uncertainties listed above, what types of uncertain results **do** you report?
    1. Do you feedback/report VUS?
    2. Do you feedback/report secondary/incidental findings, if so which would you and which would you not report?
13. Which types **do you not** report/feedback?
14. Who decides what results to report and what results not to report? The clinician? The clinical scientist/laboratory geneticist? The patient? Professional guidelines?
    1. Do you report everything you find and leave it to clinicians to decide what to feed back to the patient?
15. [Clinical scientists] Which variant classification protocols do you use? E.g. ACMG?
16. [Clinical scientists] How do you/does your lab deal with results that have some element of uncertainty?
    1. Do you purposely ‘mask’ results that might have some element of uncertainty?
17. Do you think WES/WGS raises uncertainties that are different from the uncertainties associated with CMA? If so, in what way?
18. Do you think prenatal WES/WGS/CMA raises uncertainties that are different from uncertainties associated with postnatal WES/WGS/CMA?

## Professional guidelines

1. Are you familiar with any professional guidelines for reporting prenatal CMA test results?
2. Are you familiar with any professional guidelines for reporting prenatal exome sequencing results?
3. In your experience do you believe that the current guidelines for reporting of uncertain prenatal test results is adequate or inadequate?
4. What other resources do you use when analysing results e.g. ClinVar?

###### Pre-test counselling [Healthcare practitioners]

1. What do you think are the most important aspects of a test that women (and partners) want to know about when deciding about a prenatal test?

Prompt: time, cost, types of results, test effectiveness/accuracy

1. Do you discuss the potential for results to have some element of uncertainty during pre-test counselling?
   1. What do you discuss?
2. Do couples get a choice of whether they want to receive VUS? What about secondary findings?
3. Do they provide written consent for what results they want to receive?

###### Post-test counselling [Healthcare practitioners]

1. How do you counsel patients about a VUS result?
2. What happens after a patient has received results that are uncertain?
   1. Is there any follow-up care?
   2. Psychological support?
   3. Support groups?
   4. For those continuing pregnancy, is there any change to routine clinical care e.g. further scans? Change to birth plan?
3. In your experience, what impact do uncertain results have on patients?
4. What are the potential positive outcomes from patients receiving uncertain results?
5. What are the potential negative outcomes?

Access to testing

1. Do you think pre and post-test counselling and ordering genomic tests such as WES should be restricted to specific professional groups or should all maternity practitioners be able to do this?
   1. If you think it should be restricted, who do you think should be offering it?
2. Do you think genomics testing should be only be offered to women who have a suspected fetal abnormality on ultrasound, or should it be made available to all pregnant women if they are willing to pay?

Returning uncertain results

1. **Pathogenicity and VUSs**

*How important is pathogenicity when deciding whether or not to return a result? Do you think we should only return likely or highly likely pathogenic variants? What about VUS? What about variants that are definitely or likely not pathogenic?*

1. **Incidental findings**

*Should we report incidental findings related to the baby that we might find? Which ones should we report? Which ones should we not report?*

1. **Penetrance of variant**

*Do you think we should return variants where the penetrance is not 100%?* *Do you think there should be a cut-off? If so where? 50%, 25%, 5%?*

1. **Expression of the variant**

*Do you think we should return variants that have variable expressivity? Are there any instances where you think it would not be appropriate to return a variant finding?*

1. **Diagnostic yield**

*Currently the literature reports that the chance of finding an answer for prenatal WES and WGS is around 20-40%. Do you think we should offer tests where the likelihood of finding is in this bracket? What is an acceptable limit? What if the likelihood was lower? What is unacceptable?*
